# Integration of plastic surgery into the undergraduate medical curriculum: the Norwich model and experience

**DOI:** 10.5116/ijme.4f0c.b55b

**Published:** 2012-01-25

**Authors:** Athena Au, Jong B. Kim

**Affiliations:** 1 Department of Plastic and Reconstructive Surgery, The Norfolk and Norwich University Hospital NHS Foundation Trust, UK; 2 Biomedical Research Centre, Norwich Medical School /Faculty of Medicine and Health Sciences, The University of East Anglia, UK

## Introduction

Plastic surgeons operate throughout the body, treating the widest range of trauma, malignancy, and congenital disease of any specialty. It also deals with a broad spectrum of sensitive emotional and psychological issues such as cancer diagnosis, reconstruction following disfiguring cancer and/or trauma surgery as well as difficult aesthetic issues. Therefore good communication skills and excellent doctor-patient relationships are crucial aspects of the specialty.Plastic surgery is not part of the curriculum in most medical degrees in the United Kingdom. A recent survey of UK medical schools revealed that plastic surgery was part of the curriculum in 12 out of 17 (71%) schools; but a compulsory component in only 2 schools.^1^ At the University of East Anglia School of Medicine, Health Policy and Practice (UEA) and the Norfolk and Norwich University Hospital (NNUH), the development of an Academic Plastic Surgical Unit has highlighted these issues. The UEA offers a highly innovative, fully integrated 5-year undergraduate MBBS degree programme based on the Problem Based Learning structure. It promotes learning through small group sessions supported by whole class discussions, lectures and seminars as well as self-directed learning. Within each year, clinical experience is provided in both primary care and in hospitals. This unusual modern educational approach emphasises early and frequent patient contact with the application of basic science to clinical problems right from the first year. The stated aims of the course are to produce doctors with sound medical knowledge, exceptional clinical and communication skills - the professionals who are fit for purpose to work in the twenty-first century.

### The case for plastic and reconstructive surgery

Plastic surgery has arguably the highest trauma workload including upper limb and hand injuries, soft tissue injury/loss and reconstructive surgery following trauma. It also deals with a wide range of pathologies; congenital, neoplastic to traumatic. For example, skin cancers, the commonest cancer in the western world has an increasing incidence; at least 100,000 cases are diagnosed each year, compared to 84,500 in 2007.^2^Thus, the value of teaching plastic surgery in the undergraduate curriculum is indisputable.

### Public and student perspectives

The false identity of plastic surgery perceived by the general public and medical students is a pressing concern. A recent survey amongst 1567 people, including members of the public, general practitioners and medical students, see plastic surgery to associate with reconstructive procedures following trauma or cancer and procedures with a strong-aesthetic element.1 Another survey showed 85% of the final-year students could not list 5 conditions treated by plastic surgeons2; 93% felt cosmetic surgery was the core component of the specialty.^3^ Another student survey showed 64% of students acquired their main knowledge of the specialty from the media/television.^4^ After implementing a teaching programme locally, 100% of the students could list the 5 conditions treated by plastic surgeons; 100% realised cosmetic surgery was only a small branch of the specialty. This major concern is the main driving force for this model.With the current direction of early specialisation and focused training, we feel it is essential to integrate plastic surgery into undergraduate teaching to provide students a clear identity and knowledge of the specialty. Hence, they will be able to make an informed career choice. One survey showed 60% of students felt they would be less competent without plastic surgery teaching.^4^The student survey done at the UEA found 78.0% of the students questioned wanted formal plastic surgery teaching. Less than 5% of the students found plastic surgery interesting; however irrelevant to them. The reason is that there are no plastic surgery questions in their undergraduate exam. Currently, written multiple choice questions are in the process of being added to current examination paper in the Blood and Skin module. One particular student’s comments were: ‘Do it the Mr. Kim way- interesting, my first experience of scrubbing. The extra theatre time made me realise what plastic surgery was!’The absence of formal plastic surgery teaching has led to the development of self-funding, commercial courses and conferences for students to learn about Plastic Surgery [e.g. myplasticsurgerycourse.org; undergraduate plastic reconstructive aesthetic surgery (UPRAS)]. It is unacceptable for students to have to pay for experience of a specialty that provides an important management option for a wide range of patients. However, there is a perennial problem of overcrowding of the curriculum.

### The original UEA curriculum

In the first year, students start the 5 –year programme with history taking, communication skills followed by learning the musculoskeletal system (the Locomotion Module). The second year is comprised of haematology, dermatology (the Blood and Skin module), the cardiovascular and respiratory systems. Further systems including the homeostasis and endocrinology module, the senses module and nutrition and digestion module are educated in the third year. The fourth year modules include reproduction, growth and development, and elective. Psychiatry-the Mind, emergency care and a junior-doctor shadowing rotations complete the 5-year programme in the final year. Within each module 54% of the time is devoted to theoretical studies, 13% to a clinical placement in primary care and 33% in secondary care in hospital.

### What we have done

Our solution was to integrate plastic and reconstructive surgery into the existing modules within the 5-year programme at the UEA. Presently, plastic surgery is integrated into modules Blood and Skin, and Reproduction. The advantage of the integrated UEA curriculum is to enable students to immediately apply the knowledge learnt in theoretical studies to the clinical attachments in skin cancer clinics (plastic surgery/dermatology), and plastic surgery day surgery lists. To consolidate their learning on wound management, students shadow the tissue viability nurses in treating various types of wounds. Examples of wound infections, wound healing and skin grafts etc are seen during formal bedside teaching.In the Reproduction module, their knowledge on common breast pathologies and the multidisciplinary approach to management of breast cancer are consolidated during their attachments in outpatient clinics, operating theatre and PBL discussion groups. Breast outpatient clinics would enable students to exercise the communication skills learnt in the exploration of patients’ psychosocial ideas and the art of breaking bad news. This module also introduces the realms of breast reconstructive surgery and revisits the reconstructive ladder. Operative opportunities such as assisting in mastectomies, lymph node dissections and breast reconstruction with regional or free flaps fortify their knowledge. By the end of the module, students fully appreciate the key ethos of plastic surgery - restoring form and function, and acquire a comprehensive knowledge of breast disease, management and reconstructive options.

### Our future plans

With the introduction of a full time academic plastic surgeon in the form of a clinical lecturer into the medical faculty at UEA, we are exploring the further possibility of integrating plastic surgery into other modules. Our key objectives are to increase the students’ understanding and exposure to the speciality at an early stage, provide knowledge of career opportunities, improve professional and public perception of the specialty and provide hands on surgical technical learning.We are in the process of incorporating congenital hand deformities, limb development and surgical management of rheumatoid or osteoarthritis in the locomotion module in year 1. Students would shadow the physiotherapists and assist in emergency plastic theatres to get a real essence of complex soft tissue trauma.In year 3, skin cancers, head and neck cancers are added to the senses module. Students would participate in minor operating theatres for simple excision of skin lesions as well as major reconstructive surgeries of head and neck cancers, for instance mandibulectomy with free fibular flap reconstruction to stimulate learning.Moreover, we aim to introduce common congenital deformities relating to plastic surgery such as cleft lip/palate and hypospadias in the growth and development module in the fourth year. Students would encounter such fascinating conditions in our outpatient clinics and operating lists, which are less commonly seen in other medical specialities.Additionally, plastic subspecialties such as burn and hand surgery could be naturally built-into the final year modules: Emergency Care and the Mind. The casualty is a good place for students to learn basic wound management and practise their suturing skills. Important topics such as basic hand examination, hand trauma and hand infections would be taught in the emergency care module. Burns injuries are very commonly seen in casualty. It is important to educate students on the basic knowledge in assessing the type, the degree and estimate the surface area of burns, and subsequently formulating the initial management of burns. Furthermore, burns and body dysmorphic disorders could be integrated nicely in the Mind module. Students could gain insight into the psychosocial aspects and the challenges of reconstructive surgery in these conditions.

## Conclusions

We present our experience on the integration of plastic surgery into the UEA undergraduate medical curriculum. The fully integrated teaching approach at the UEA provides an example of how plastic surgery can be taught throughout the undergraduate degree. The benefits of plastic surgery to medical students include, gaining insight into the various diseases, workload and patient care involving plastic surgery, acquiring the skills to diagnosis and manage common plastic surgery-related injuries and enabling students with an interest in plastic surgery to make an informed career decision at an early stage. Subsequently, students can plan to further their experiences of the specialty through plastic surgical placements at electives, foundation training, basic surgical training, courses and research projects.We hope this will enlighten medical faculties in other medical schools worldwide with a fully integrated medical course to consider encompassing Plastic and Reconstructive surgery into their curriculum similar to that of University of East Anglia.

### Conflict of Interest

The authors declare that they have no conflict of interest.
